# Prevalence of arterial hypertension in Brazilian adults and its associated factors and activity limitations: a cross-sectional study

**DOI:** 10.1590/1516-3180.2018.0251220719

**Published:** 2019-10-31

**Authors:** Aline Pinto Marques, Célia Landmann Szwarcwald, Paulo Roberto Borges de, Déborah Carvalho Malta, Dalia Elena Romero Montilla

**Affiliations:** I PhD. Assistant Researcher, Laboratory of Health Information, Institute of Health Communication and Scientific and Technological Information, Fundação Oswaldo Cruz (Fiocruz), Rio de Janeiro (RJ), Brazil.; II PhD. Researcher, Laboratory of Health Information, Institute of Health Communication and Scientific and Technological Information, Fundação Oswaldo Cruz (Fiocruz), Rio de Janeiro (RJ), Brazil.; III PhD. Research Assistant, Laboratory of Health Information, Institute of Health Communication and Scientific and Technological Information, Fundação Oswaldo Cruz (Fiocruz), Rio de Janeiro (RJ), Brazil.; IV PhD. Professor, School of Nursing, Universidade Federal de Minas Gerais (UFMG), Belo Horizonte (MG), Brazil.; V PhD. Researcher, Laboratory of Health Information, Institute of Health Communication and Scientific and Technological Information, Fundação Oswaldo Cruz (Fiocruz), Rio de Janeiro (RJ), Brazil.

**Keywords:** Hypertension, Blood pressure, Risk factors, Socioeconomic factors

## Abstract

**BACKGROUND:**

Hypertension is a serious global public health problem that affects a large part of the Brazilian adult population and can cause limitations and losses of quality of life.

**OBJECTIVE:**

The objective of this study was to analyze the association of hypertension and its correlated limitations, with sociodemographic and epidemiological factors.

**DESIGN AND SETTING:**

Cross-sectional study analyzing information on 44,271 adults (30 years or older) from the Brazilian National Health Survey of 2013.

**METHODS:**

The prevalence of hypertension and the degree of limitation of the patients’ activities associated with hypertension, according to sociodemographic characteristics, anthropometric measurements and lifestyles, were calculated for both sexes. To analyze the strength of association, bivariate and multivariate Poisson regression were used.

**RESULTS:**

Hypertension was the most prevalent risk factor among Brazilian adults aged 30 years or older (40.7%). It was strongly associated with the aging process (prevalence ratio, PR 3.51), obesity (PR 1.73), heart disease (PR 1.67) and stroke (PR 1.86). Furthermore, limitations associated with hypertension were more prevalent among those with comorbidities from noncommunicable diseases relating to hypertension complications (stroke PR 1.47; heart disease PR 1.69) and with incomplete elementary education (PR 1.19).

**CONCLUSIONS:**

This study showed sociodemographic inequality in the prevalence of hypertension, especially in the population with some degree of limitation associated with hypertension. It showed that improvements in access to primary care services for controlling hypertension at its initial stages are essential in order to avoid comorbidities of greater severity and limitations and losses of quality of life, especially among socially disadvantaged people.

## INTRODUCTION

According to a World Health Organization (WHO) report,^1^ noncommunicable diseases (NCDs) were responsible for 38 million deaths in 2012, thus accounting for 70% of deaths worldwide. Hypertension is considered to be an intermediate risk factor, given its substantial impact on the burden of cardiovascular diseases.^2^^,^^3^ WHO has estimated that, in 2008, around 12.8% of the deaths resulted from hypertension.^4^ Moreover, it has been estimated that around 40% of the adult population worldwide aged 24 years or older has hypertension, and that this proportion is in even higher in low and middle-income countries in which living conditions are worse and the healthcare systems are fragile.^5^

The relationship between hypertension and both cardiovascular and cerebrovascular diseases is well known, stable and independent of other risk factors.^6^ Arterial hypertension is recognized as a major public health problem that contributes greatly to increased burdens of cardiopathies, stroke, renal failure and disability.^2^

Hypertension is an important risk factor among the Brazilian adult population. According to PNAD 2008 data, nearly 14% of Brazilians aged 18 years or over self-reported having a diagnosis of hypertension.^7^ This proportion has increased over more recent years. In 2013, the prevalence of self-reported hypertension in the same population was 32.3%, with a sharp increase among adults aged 30 years and over.^8^

Hypertension has complex and multifactorial characteristics, and it combines hereditary and genetic factors with socioeconomic, environmental and lifestyle factors. Knowing the risk factors associated with hypertension, along with the magnitudes of the associations, is fundamental for making it possible to reduce hypertension and the morbidity and mortality caused by other NCDs.

## OBJECTIVE

The objective of the present study was to analyze the association of hypertension and the limitations associated with this, with sociodemographic and epidemiological factors, among the Brazilian adult population aged 30 years or over.

## METHODS

### Study design, sampling and ethics

This cross-sectional study analyzed the prevalence of hypertension and the degree of limitation of the patients’ activities associated with hypertension, according to information from the Brazilian National Health Survey (Pesquisa Nacional de Saúde, PNS) of 2013. PNS 2013 was a household survey on the Brazilian population according to major regions, states and state capitals, using a representative sample design. The foremost objective of this survey was to characterize the health situation of the Brazilian population and its lifestyles, and to collect information about its healthcare and access to and utilization of healthcare services.

The PNS had a complex sampling design. Three-stage cluster sampling (census tracts, households and individuals) was used, with stratification of the primary sampling units (PSUs) and random selection in each stratum. Census tracts or sets of sectors composed the PSUs; households were the units of the second stage; and residents aged 18 years or older defined the third-stage unit.^9^ The PNS fieldwork was carried out from August 2013 to February 2014, in 6,069 selected census tracts, with visits to 81,254 homes. Among these, 69,994 homes were found to be occupied, 64,348 household interviews were conducted and 60,202 individual interviews were completed. In this study, we restricted the data analysis to adults aged 30 years and older (44,271 individuals).

This study was approved by the National Commission for Research Ethics (Comissão Nacional de Ética em Pesquisa, CONEP) in June 2013 (No. 328,159).^10^

### Study outcomes and explanatory variables

The hypertension definition used was that subjects were classified as hypertensive when their final systolic blood pressure (average of three measurements) was greater than or equal to 140 mmHg and/or their final diastolic blood pressure was greater than or equal to 90 mmHg and/or they self-reported having used antihypertensive medication in the last two weeks. The methods of assessment and the definition of hypertension were based upon the recommendation of the evidence-based guideline for the management of high blood pressure in adults: report from the panel members appointed to the Eighth Joint National Committee (JNC8).^6^

The limitations associated with hypertension were defined as including only the individuals with a self-reported diagnosis of hypertension with some degree of limitation (responses 2 to 4), based on the following question: “In general, to what extent does hypertension or some complication of hypertension limit your usual activities (such as working, studying, doing household chores, etc.)? 1. none; 2. a little; 3. greatly; or 4. very greatly”.

The sociodemographic characteristics used in the analysis were: sex (male or female); age group (30 to 39 years, 40 to 49 years, 50 to 59 years or 60 years or over); race/color (white, black or brown/mixed); and educational level (elementary education incomplete; elementary education completed or high school education incomplete; or high school education completed or more).

The following anthropometric variables, both classified according to WHO recommendations, were considered in the analysis: body mass index (BMI) (< 25 kg/m², 25-29 kg/m² or ≥ 30 kg/m²); and waist circumference (WC) (women: < 80 cm normal; 80-87.9 cm enlarged; or ≥ 88 cm substantially enlarged; and men: < 94 cm normal; 94-101.9 cm enlarged; or ≥ 102 cm substantially enlarged).^10^

We also considered variables representing lifestyles: smoking (current smoker, former smoker or nonsmoker); recommended daily consumption of fruit and vegetables on five or more days per week (yes or no); and recommended physical activity in leisure time greater than or equal to 150 minutes per week (yes or no).

The health situation characteristics assessed included the following variables regarding health status and access to healthcare services: self-rated health (good/very good; or fair/poor/very poor); chest pain when walking (yes or no); self-reported diagnosis of some heart disease (yes or no); self-reported stroke (yes or no); self-reported diabetes (yes or no); depression based on the Patient Health Questionnaire-9 (PHQ-9)^11^ (yes or no) and at least one medical consultation in the last 12 months (yes or no).

To ensure the quality of information and control for potential bias, we trained the interviewers; experienced researchers prepared an operational manual for interviewers; and the anthropometric and blood pressure measurements followed international protocols. A pilot test was conducted in March 2013 and fieldwork coordination was carried out by the Brazilian Institute for Geography and Statistics (IBGE).^10^

### Statistical analysis

To study the association between hypertension and sociodemographic characteristics, lifestyle factors and health status, we estimated the hypertension prevalence ratio (PR) and 95% confidence intervals (95% CI), using Poisson regression models. The outcome was the presence of hypertension and the independent variables were the following: sex, age group, race/skin color, educational level, BMI, WC, smoking, adequate consumption of fruits and vegetables, being physically active, self-rated health, chest pain when walking, heart disease, stroke, diabetes, depression based on PHQ-9 and at least one medical consultation in the last year.

The statistical analysis was done separately for four groups of variables: sociodemographic characteristics; anthropometric measurements; lifestyles; and health situation. For each group of variables, we calculated the crude prevalence ratio. Then, we calculated the adjusted prevalence ratio according to sex and age (model 1) and according to all other covariates (model 2).

The data were analyzed using the Stata software, version 11.0, through the survey module, which incorporates the effects of complex samples.

## RESULTS

In 2013, the prevalence of hypertension in the Brazilian population aged 30 years or older was 40.7%. The prevalence of hypertension was seen to increase with age: while the prevalence was only 18.8% in the age group from 30 to 39 years, the rate reached 66% in the age group of 60 years or older. People with lower educational level and of black race/color had higher prevalence of hypertension (Table 1).

**Table 1.** Prevalence of hypertension and some degree of limitation associated with this among adults aged 30 years or older, according to sociodemographic, anthropometric, lifestyle and health status characteristics (n = 44,271)

	n	%	Hypertension		Some degree of limitation¹	
			Prevalence ratio	95% CI	Prevalence ratio	95% CI
Total	44,271	100.0	40.7	(39.8-41.5)	30.8	(29.3-32.4)
Sociodemographic characteristics						
Age						
30 to 39	12,811	28.9	18.8	(17.7-20.0)	28.3	(24.3-32.7)
40 to 49	10,851	24.5	33.1	(31.5-34.6)	28.1	(24.9-31.6)
50 to 59	9,742	22.0	49.6	(47.7-51.4)	30.5	(27.7-33.4)
60 or older	10,866	24.5	66.0	(64.5-67.6)	32.8	(30.6-35.0)
Sex						
Male	20,575	46.5	41.0	(39.8-42.3)	26.1	(24.0-28.4)
Female	23,696	53.5	40.3	(39.3-41.4)	34.0	(32.0-36.0)
Education level						
High school education or over	17,966	40.6	31.4	(30.1-32.6)	19.1	(16.9-21.5)
Elementary education completed/high school education incomplete	5,655	12.8	37.6	(35.4-39.9)	29.0	(25.0-33.3)
Elementary education incomplete	20,649	46.6	49.6	(48.4-50.8)	37.4	(35.4-39.5)
Race/color						
White	21,629	48.9	41.2	(40.0-42.5)	27.6	(25.6-29.7)
Black	4,189	9.5	45.0	(42.4-47.6)	36.1	(31.4-41.2)
Brown/mixed	17,861	40.3	39.1	(37.9-40.3)	33.8	(31.4-36.3)
**Anthropometric and lifestyle characteristics**						
Body mass index						
< 25 kg/m²	16,232	36.7	31.3	(30.1-32.6)	32.0	(29.2-34.9)
25-29 kg/m²	17,447	39.5	41.1	(39.8-42.5)	30.3	(28.0-32.7)
≥ 30 kg/m²	10,509	23.8	54.2	(52.5-55.9)	30.7	(28.1-33.5)
Waist circumference^²^						
Normal	14,371	32.5	27.4	(26.1-28.7)	32.9	(29.5-36.4)
Enlarged	9,908	22.4	36.2	(34.6-37.8)	29.1	(26.0-32.3)
Substantially enlarged	19,991	45.2	52.4	(51.1-53.7)	30.8	(28.9-32.7)
Smoking						
Smoker	7,077	16.0	40.2	(38.0-42.4)	31.8	(28.1-35.7)
Former smoker	9,292	21.0	51.4	(49.7-53.1)	31.5	(28.8-34.3)
Nonsmoker	27,902	63.0	37.2	(36.2-38.3)	30.3	(28.3-32.3)
Recommended consumption of fruit and vegetables						
No	30,762	69.5	40.8	(39.8-41.8)	32.3	(30.4-34.2)
Yes	13,509	30.5	40.4	(39.8-41.8)	27.5	(24.9-30.3)
Physical activity during free time						
No	36,094	81.5	42.1	(41.2-43.0)	32.7	(31.1-34.4)
Yes	8,176	18.5	34.5	(32.7-36.2)	20.7	(17.8-24.1)
**Health situation characteristics**						
Self-rated health						
Good/very good	26,791	60.5	33.2	(32.1-34.2)	17.4	(15.4-19.6)
Fair/poor/very poor	17,479	39.5	52.1	(50.8-53.4)	39.9	(37.9-41.9)
Chest pain when walking						
No	38,486	86.9	38.8	(37.9-39.7)	24.8	(23.3-26.4)
Yes	5,784	13.1	53.1	(50.9-55.4)	52.3	(48.8-55.8)
Some heart disease						
No	41,892	94.6	39.3	(40.1-40.7)	28.4	(26.8-30.1)
Yes	2,378	5.4	65.5	(61.7-69.1)	48.1	(43.4-52.8)
Stroke						
No	43,370	98.0	40.0	(39.1-40.8)	30.1	(28.5-31.7)
Yes	901	2.0	74.4	(69.4-78.8)	44.1	(37.6-50.9)
Diabetes						
No	36,729	90.9	38.4	(37.5-39.3)	28.3	(26.6-30.0)
Yes	3,656	9.1	73.2	(70.6-75.8)	40.3	(36.7-44.0)
Depression (PHQ-9 scale)						
No	39,465	89.1	39.8	(39.0-40.7)	26.7	(25.1-28.4)
Yes	4,806	10.9	47.4	(44.9-50.0)	51.8	(48.2-55.3)
Medical appointment within the last 12 months						
No	10,095	23.0	32.0	(30.4-33.7)	20.9	(17.5-24.7)
Yes	33,887	77.0	43.3	(42.3-44.3)	31.9	(30.3-33.6)

1) Self-reported hypertension; 2) Female (normal: < 80 cm; enlarged: 80-87.9 cm; substantially enlarged: ≥ 88 cm); Male (normal: < 94 cm; enlarged: 94-101.9 cm; substantially enlarged: ≥ 102 cm). Source: National Health Survey, 2013.^9^ CI = confidence interval; PHQ-9 = Patient Health Questionnaire-9.

The results presented in Table 1 show that the higher the BMI was, the larger the prevalence of hypertension also was, reaching 54.2% among obese individuals (those with BMI greater than or equal to 30 kg/m²). The same results were found for WC: among people with substantially enlarged WC, the prevalence of hypertension was 52.4%. Regarding the habit of smoking, former smokers and current smokers showed higher prevalence of hypertension than nonsmokers did. The prevalence of hypertension was higher among physically inactive individuals.

With regard to health situation (Table 1), the prevalence of hypertension among people with fair/poor self-reported health was higher (52.1%). People with other NCDs also showed high prevalence rates: 65.5% among those with heart diseases, 74.4% among those who had had a stroke, 73.2% among those with diabetes and 47.4% among people with depression.

Analysis on the presence of limitations associated with hypertension (Table 1) showed that 69.2% of the hypertensive individuals did not have any limitations associated with the disease. Women reported limitations associated with hypertension more frequently than did men: 34.0% and 26.1%, respectively. Elderly people reported limitations associated with hypertension to a higher degree than did individuals aged 30-39 years. Significant differences in the prevalence of limitations associated with hypertension were found according to educational level: the lower the degree of education was, the higher the percentage of people with some limitations was. The prevalence of these limitations ranged from 19.1% to 37.4%. White people (27.6%) reported lower limitations than did black people (36.1%) or brown/mixed-race people (33.8%).

People with high BMI and high WC presented slightly lower proportions of limitations. There was also no statistical difference in the limitations associated with hypertension levels according to smoking status. On the other hand, people with adequate consumption of fruits and vegetables had lower limitations (72.5% had no limitations associated with hypertension) and most of the physically active individuals with hypertension reported that they did not have any limitations associated with the disease (79.3%).

Limitations associated with hypertension were more common among individuals with worse health status. Among those with fair/poor self-reported health, 39.9% reported having some limitations. More than half (52.3%) of those who reported having chest pain when walking also reported some limitations. Among people with other NCDs, the prevalence of limitations associated with hypertension was also high: 48.1% for another heart disease; 44.1% for stroke; 40.3% for diabetes; and 51.8% for depression based on PHQ-9. Among individuals who had had at least one medical consultation in the last 12 months, 31.9% reported having some limitations associated with hypertension (Table 1).

Table 2 shows the association analysis for sociodemographic characteristics in relation to hypertension and the limitations associated with hypertension. Greater age was strongly associated with greater hypertension, such that the prevalence ratio (PR) among elderly people (≥ 60 years) was three times higher than that of the youngest age group (30-39 years). Lower educational levels were also associated with higher prevalence of hypertension, in all the regression models considered, especially for individuals with incomplete elementary school in comparison with those with completed high school or more (PR 1.19; 95% CI 1.14-1.25). Black individuals showed higher prevalence of hypertension in all models, in comparison with white individuals.

**Table 2.** Results from Poisson regression models in which hypertension and some degree of limitation associated with this were the outcomes and sociodemographic characteristics were the covariates, among adults aged 30 years or older (n = 44,271)

Hypertension	Unadjusted model			Model 1¹			Model 2²		
	PR	95% CI	P-value	PR	95% CI	P-value	PR	95% CI	P-value
Age									
30-39	1			1			1		
40-49	1.76	(1.63-1.89)	< 0.001	1.76	(1.63-1.89)	< 0.001	1.72	(1.60-1.85)	< 0.001
50-59	2.64	(2.45-2.83)	< 0.001	2.64	(2.45-2.83)	< 0.001	2.54	(2.36-2.73)	< 0.001
60+	3.51	(3.29-3.75)	< 0.001	3.52	(3.29-3.76)	< 0.001	3.29	(3.07-3.53)	< 0.001
Sex									
Female	1			1			1		
Male	1.02	(0.98-1.06)	0.398	1.04	(1.00-1.08)	0.024	1.04	(1.00-1.08)	0.036
Education level									
High school education or over	1			1			1		
Elementary education completed/high school education incomplete	1.20	(1.11-1.29)	< 0.001	1.12	(1.04-1.20)	0.002	1.11	(1.04-1.20)	0.003
Elementary education incomplete	1.58	(1.50-1.65)	< 0.001	1.19	(1.14-1.25)	< 0.001	1.19	(1.14-1.25)	< 0.001
Race/color									
White	1			1			1		
Black	1.09	(1.02-1.16)	0.008	1.12	(1.05-1.19)	< 0.001	1.09	(1.02-1.16)	0.007
Brown/mixed	0.95	(0.91-0.99)	0.014	1.02	(0.98-1.06)	0.351	0.99	(0.94-1.03)	0.565
**Some degree of limitation³**	**Unadjusted model**			**Model 1¹**			**Model 2²**		
	**PR**	**95% CI**	**P-value**	**PR**	**95% CI**	**P-value**	**PR**	**95% CI**	**P-value**
Age									
30-39	1			1			1		
40-49	0.99	(0.83-1.18)	0.937	0.99	(0.83-1.18)	0.907	0.94	(0.79-1.11)	0.458
50-59	1.08	(0.91-1.28)	0.399	1.08	(0.91-1.28)	0.400	0.99	(0.84-1.18)	0.925
60+	1.16	(0.98-1.37)	0.084	1.15	(0.98-1.36)	0.095	0.96	(0.81-1.13)	0.622
Sex									
Female	1			1			1		
Male	0.77	(0.70-0.85)	< 0.001	0.77	(0.70-0.85)	< 0.001	0.81	(0.73-0.89)	< 0.001
Schooling level									
High school education or over	1			1			1		
Elementary education completed/high school education incomplete	1.50	(1.25-1.80)	< 0.001	1.50	(1.25-1.80)	< 0.001	1.47	(1.22-1.76)	< 0.001
Elementary education incomplete	1.96	(1.72-2.22)	< 0.001	1.94	(1.71-2.20)	< 0.001	1.89	(1.66-2.15)	< 0.001
Race/color									
White	1			1			1		
Black	1.31	(1.13-1.52)	< 0.001	1.31	(1.13-1.52)	< 0.001	1.23	(1.05-1.42)	0.008
Brown/mixed	1.22	(1.11-1.35)	< 0.001	1.23	(1.11-1.36)	< 0.001	1.13	(1.02-1.25)	0.016

1) Model 1 = sex and age; 2) Model 2 = all variables; 3) Self-reported hypertension. Source: National Health Survey, 2013.^9^ PR = prevalence ratio; CI = confidence interval.

Regarding the presence of limitations associated with hypertension, the variables associated were sex, educational level and race/color. Women showed higher prevalence of limitations associated with hypertension in all regression models. The lowest level of schooling (incomplete elementary school) was strongly associated with the presence of limitations associated with hypertension. Not being white was also significantly associated (Table 2).

The results presented in Table 3 show that high BMI or WC were significantly associated with hypertension in all the models analyzed. Among people with substantially enlarged WC, the PR was 1.91 (95% CI 1.18-2.02) in the bivariate analysis and 1.53 (95% CI 1.43-1.64) in the multivariate analysis. Both the crude analysis and model 1 (adjusted according to sex and age) showed that physically inactive people had higher prevalence of hypertension, but the association was not significant in the multivariate model. Hypertension did not show any association with consumption of fruits and vegetables or with smoking in any of the models. However, the probability of being hypertensive was higher among former smokers.

**Table 3.** Results from Poisson regression models in which hypertension and some degree of limitation associated with this were the outcomes and anthropometric and lifestyle indicators were the covariates, among adults aged 30 years or older (n = 44,271)

Hypertension	Unadjusted model			Model 1¹			Model 2²		
	PR	95% CI	P-value	PR	95% CI	P-value	PR	95% CI	P-value
Body mass index									
< 25 kg/m²	1			1			1		
25-29 kg/m²	1.31	(1.22-1.38)	< 0.001	1.28	(1.22-1.35)	< 0.001	1.09	(1.03-1.16)	0.003
≥ 30 kg/m²	1.73	(1.64-1.82)	< 0.001	1.68	(1.60-1.76)	< 0.001	1.30	(1.22-1.38)	< 0.001
Waist circumference³									
Normal	1			1			1		
Enlarged	1.32	(1.23-1.41)	< 0.001	1.30	(1.23-1.38)	< 0.001	1.24	(1.17-1.33)	< 0.001
Substantially enlarged	1.91	(1.81-2.02)	< 0.001	1.77	(1.67-1.86)	< 0.001	1.53	(1.43-1.64)	< 0.001
Smoking									
Nonsmoker	1			1			1		
Smoker	1.08	(1.02-1.15)	0.012	1.01	(0.95-1.08)	0.680	1.05	(0.99-1.12)	0.085
Former smoker	1.38	(1.32-1.44)	< 0.001	1.12	(1.08-1.17)	< 0.001	1.08	(1.03-1.13)	< 0.001
Recommended consumption of fruit and vegetables									
No	1			1			1		
Yes	0.99	(0.95-1.03)	0.633	0.97	(0.93-1.01)	0.121	1.00	(0.96-1.04)	0.810
Physical activity during free time									
Yes	1			1			1		
No	1.22	(1.16-1.29)	< 0.001	1.10	(1.04-1.16)	< 0.001	1.05	(1.00-1.11)	0.056
**Some degree of limitation^4^**	**Unadjusted model**			**Model 1¹**			**Model 2²**		
	**PR**	**95% CI**	**P-value**	**PR**	**95% CI**	**P-value**	**PR**	**95% CI**	**P-value**
Body mass index									
< 25 kg/m²	1			1			1		
25-29 kg/m²	1.06	(0.94-1.19)	0.347	0.95	(0.85-1.07)	0,382	1,08	(0.95-1.22)	0.239
≥ 30 kg/m²	1.01	(0.90-1.33)	0.799	0.95	(0.84-1.08)	0.457	1.12	(0.96-1.30)	0.155
Waist circumference³									
Normal	1			1			1		
Enlarged	0.88	(0.76-1.03)	0.112	0.84	(0.73-0.98)	0.029	0.86	(0.74-1.00)	0.051
Substantially enlarged	0.94	(0.83-1.06)	0.285	0.83	(0.73-0.94)	0.004	0.80	(0.69-0.93)	0.004
Smoking									
Nonsmoker	1			1			1		
Smoker	1.05	(0.91-1.20)	0.498	1.11	(0.97-1.27)	0.133	1.02	(0.89-1.16)	0.793
Former smoker	1.04	(0.93-1.16)	0.478	1.09	(0.97-1.21)	0.134	1.05	(0.95-1.17)	0.325
Recommended consumption of fruit and vegetables									
No	1			1			1		
Yes	0.85	(0.76-0.95)	0.005	0.84	(0.76-0.94)	0.003	0.91	(0.82-1.02)	0.101
Physical activity during free time									
Yes	1			1			1		
No	1.58	(1.35-1.84)	< 0.001	1.56	(1.34-1.82)	< 0.001	1.38	(1.19-1.61)	< 0.001

1) Model 1 = sex and age; 2) Model 2 = sex, age, anthropometric and lifestyle variables; 3) Female (normal: < 80 cm; enlarged: 80-87.9 cm; substantially enlarged: ≥ 88 cm); Male (normal: < 94 cm; enlarged: 94-101.9 cm; substantially enlarged: ≥ 102 cm); 4) Self-reported hypertension. Source: National Health Survey, 2013.^9^ PR = prevalence ratio; CI = confidence interval.

Regarding some reported limitations associated with hypertension (Table 3), neither BMI nor smoking habits showed any association with the outcome. On the other hand, physical inactivity during leisure time was strongly associated with the limitations linked with hypertension. in all the analysis models, with PR ranging from 1.58 (95% CI 1.35-1.84) in the bivariate model to 1.38 (95% CI 1.19-1.61) in model 2. Individuals with enlarged WC had slightly lower prevalence of limitations associated with hypertension in the multivariate models 1 and 2.

Table 4 shows the PR (crude and adjusted) for the association of health status with hypertension and the limitations associated with hypertension. The disease most strongly associated with hypertension was diabetes, with PR ranging from 1.91 (95% CI 1.83-1.99) in the bivariate model to 1.31 (95% CI 1.25-1.36) in multivariate model 2. Other indicators that also showed significantly higher prevalence of hypertension were the following: fair/poor/very poor self-reported health; stroke; chest pain when walking; some heart disease; and medical consultation within the last year. Depression was associated with hypertension in the bivariate model and in model 1.

**Table 4.** Results from Poisson regression models in which hypertension and some degree of limitation associated with this were the outcomes and health situation indicators were the covariates, among adults aged 30 years or older (n = 44,271)

Hypertension	Unadjusted model			Model 1¹			Model 2²		
	PR	95% CI	P-value	PR	95% CI	P-value	PR	95% CI	P-value
Self-rated health									
Good/very good	1			1			1		
Fair/poor/ very poor	1.57	(1.51-1.63)	< 0.001	1.26	(1.21-1.31)	< 0.001	1.18	(1.13-1.23)	< 0.001
Chest pain when walking									
No	1			1			1		
Yes	1.37	(1.31-1.43)	< 0.001	1.25	(1.19-1.30)	< 0.001	1.16	(1.11-1.21)	< 0.001
Some heart disease									
No	1			1			1		
Yes	1.67	(1.57-1.77)	0.004	1.26	(1.19-1.34)	0.004	1.10	(1.03-1.17)	0.004
Stroke									
No	1			1			1		
Yes	1.86	(1.74-1.99)	< 0.001	1.33	(1.24-1.41)	< 0.001	1.17	(1.10-1.25)	< 0.001
Diabetes									
No	1			1			1		
Yes	1.91	(1.83-1.99)	< 0.001	1.41	(1.36-1.47)	< 0.001	1.31	(1.25-1.36)	< 0.001
Depression scale (PHQ-9 scale)									
No	1			1			1		
Yes	1.19	(1.13-1.26)	< 0.001	1.13	(1.07-1.19)	< 0.001	1.00	(0.95-1.05)	0.910
Medical appointment within the last 12 months									
No	1			1			1		
Yes	1.35	(1.28-1.43)	< 0.001	1.23	(1.16-1.29)	< 0.001	1.16	(1.10-1.23)	< 0.001
**Some degree of limitation^3^**	**Unadjusted model**			**Model 1¹**			**Model 2²**		
	**PR**	**95% CI**	**P-value**	**PR**	**95% CI**	**P-value**	**PR**	**95% CI**	**P-value**
Self-rated health									
Good/very good	1			1			1		
Fair/poor/ very poor	2.29	(2.02-2.60)	< 0.001	2.25	(1.98-2.55)	< 0.001	1.68	(1.48-1.93)	< 0.001
Chest pain when walking									
No	1			1			1		
Yes	2.11	(1.93-2.29)	< 0.001	2.09	(1.92-2.28)	< 0.001	1.59	(1.45-1.75)	< 0.001
Some heart disease									
No	1			1			1		
Yes	1.69	(1.51-1.89)	< 0.001	1.69	(1.51-1.90)	< 0.001	1.23	(1.10-1.38)	< 0.001
Stroke									
No	1			1			1		
Yes	1.47	(1.25-1.72)	< 0.001	1.47	(1.26-1.72)	< 0.001	1.20	(1.04-1.40)	0.015
Diabetes									
No	1			1			1		
Yes	1.43	(1.28-1.59)	< 0.001	1.39	(1.25-1.55)	< 0.001	1.19	(1.08-1.32)	0.001
Depression (PHQ-9 scale)									
No	1			1			1		
Yes	1.94	(1.77-2.12)	< 0.001	1.90	(1.72-2.08)	< 0.001	1.33	(1.21-1.47)	< 0.001
Medical appointment within the last 12 months									
No	1			1			1		
Yes	1.53	(1.28-1.82)	< 0.001	1.45	(1.21-1.74)	< 0.001	1.22	(1.00-1.48)	0.047

1) Model 1 = sex and age; 2) Model 2 = sex, age and health situation; 3) Self-reported hypertension. Source: National Health Survey, 2013.^9^ PR = prevalence ratio; CI = confidence interval; PHQ-9 = Patient Health Questionnaire-9.

Regarding the analysis on the association between health status and some limitations associated with hypertension, we observed that people with fair/poor self-reported health presented PR 1.68 (95% CI 1.48-1.93) after controlling for all other variables. Likewise, for chest pain when walking, the PR was 1.59 (95% CI 1.45-1.75). For some heart disease, diabetes or stroke, the PR was approximately 1.20 in the multivariate model. Depression was strongly associated with limitations relating to hypertension, with PR ranging from 1.94 (95% CI 1.77-2.12) in the bivariate analysis to 1.33 (95% CI 1.21-1.47) in the multivariate model. Having had a medical consultation within the last year showed a significant association in the bivariate analysis and in model 1, but lost significance after controlling for all other variables (Table 4).

## DISCUSSION

This study used blood pressure measurements to define hypertension, unlike other national studies that used self-reported diagnoses of hypertension.^12^^,^^13^ However, studies based on reported morbidity are impacted by inequalities in healthcare access.^14^^,^^15^ Use of blood pressure measurements allows better prevalence estimates for the outcome in the population. Nevertheless, in the case of limitations associated with hypertension, we had to consider only those individuals who had been previously diagnosed.

The results from this study showed that the prevalence of hypertension in the Brazilian adult population aged 30 years or over is high (40.7%), and that it increases progressively with age, especially among elderly individuals. The prevalence was also significantly higher among people who were nonwhite or less schooled, those with high BMI or enlarged WC, former smokers, those who were physically inactive and those who had had at least one medical consultation in the last 12 months before the survey.

Regarding comorbidities, individuals with fair/poor/very poor self-reported health, chest pain when walking, heart disease, diabetes, stroke or depression showed greater prevalence of hypertension. Regarding the limitations associated with hypertension, the prevalence was significantly higher among women, elderly people, people with lower schooling levels, nonwhites, people who were physically inactive and people consuming insufficient daily amounts of fruits and vegetables.

Male subjects presented higher prevalence of hypertension than did female subjects. This finding contrasted with the results from another study in which higher prevalence of hypertension among women was observed through use of self-reported data on diagnoses of hypertension.^13^ Use of a self-reported variable of diagnosis is influenced by the degree of knowledge of the diagnosis, which, in turn, is related to access to and use of healthcare services. It is known that Brazilian women seek healthcare services more often than men do, which thus leads to underdiagnosing of hypertension among men.^16^

It has been recognized in the literature both from Brazil and from other countries that hypertension occurs most frequently at older ages. Physiological changes occur in the cardiovascular system as a normal characteristic of the aging process.^17^ The increasing life expectancy and aging of the Brazilian population, as well as in the whole world, are factors that can explain the increase in the prevalence of hypertension over the last decades.^5^

The higher prevalence of hypertension among nonwhites that was found in the present study is in line with the findings from other studies on the Brazilian population.^8^^,^^13^ The limitations associated with hypertension were also greater among nonwhite individuals. Some studies have pointed out genetic characteristics to explain the higher prevalence of hypertension among black people.^17^ However, other factors that can be correlated with this include the accumulation of social disadvantages that have historically been found in this population group, which experiences greater socioeconomic vulnerability and lower access to healthcare services.^18^

In the present study, important socioeconomic inequalities in the distribution of hypertension were observed with regard not only to skin color, but also to educational level. Many studies have pointed out that multiple socioeconomic characteristics are associated with greater risk of hypertension, both at the individual level^19^ and at the structural level.^20^ It has been shown that individuals exposed to worse socioeconomic conditions are at greater risk of becoming hypertensive.^21^

The findings from the present study indicated that the lower the schooling level was, the greater the prevalence of hypertension and limitations associated with this were. Schooling level is an individual attribute that is generally imprinted on people’s life cycles, thus influencing their opportunities, choices and experiences. Among adult individuals, it is a relatively stable characteristic, in comparison with younger people. It has been indicated in the scientific literature that low schooling levels heighten the exposure to risk factors for hypertension, such as improper feeding, smoking, physical inactivity during leisure time and higher levels of psychosocial stress.^18^

Both anthropometric measurements that were used in the present study to define obesity were significantly associated with higher prevalence of hypertension. It is well known that obesity is a strong predictor for cardiovascular diseases.^5^ Data from the Brazilian National Health Survey showed that obesity (BMI ≥ 30 kg/m²) resulted in an increase of 5.6 mmHg in systolic blood pressure and 3.1 mmHg in diastolic blood pressure among men and, respectively, 3.8 mmHg and 2.0 mmHg among women.^22^

The GBD study pointed out that smoking was responsible for 45% of the deaths associated with acute myocardial infarction and 25% of the deaths caused by cerebrovascular diseases.^23^ In 2011, 21% of the deaths due to cardiovascular diseases and 18% due to stroke in Brazil were also associated with smoking habits.^24^ The results from the present study showed that there was an association between formerly smoking and presenting hypertension. Since PNS was a cross-sectional study, formerly smoking represented tobacco exposure in the past, with smoking cessation greater among individuals diagnosed with hypertension.^25^

All the NCDs analyzed showed associations with hypertension, except depression. This finding corroborated results in the literature regarding the relationship between hypertension and other NCDs.^26^ Comorbidities also heightened the prevalence of limitations associated with hypertension, since coexistence with other diseases can exacerbate the limiting effects.

Having had a medical consultation within the last 12 months before the survey was also associated with presence of hypertension. Greater access to healthcare services increases the opportunity to make the diagnosis, the chance of treatment and the life expectancy of individuals.^27^ Nevertheless, the rate of diagnosing hypertension is still low in Brazil. A recent study that used PNS data indicated that only 43.2% of hypertensive adults had received a diagnosis of the disease. Hypertension is a silent disease, which makes early diagnosis a major challenge in primary healthcare services.^28^

One limitation of the present study was its use of a self-reported question about limitations associated with hypertension. Poor health status or depression can increase the perception of limitations in people’s usual activities and may influence the frequency of negative responses to this question.

## CONCLUSION

Hypertension is the most prevalent risk factor among Brazilian adults aged 30 years or over. It is strongly associated with the aging process, obesity and cardiovascular and cerebrovascular diseases. Furthermore, occurrences of limitations associated with hypertension are more prevalent among individuals with comorbidities of NCDs relating to the complications of hypertension. Therefore, it is essential to make improvements in access to primary care services for controlling hypertension at its initial stages, thus avoiding comorbidities of greater severity and limitations and losses of quality of life, especially among socially disadvantaged people.
